# Use of Modified Polysaccharide 4DryField^*®*^ PH for Adhesion Prevention and Hemostasis in Gynecological Surgery: A Two-Center Observational Study by Second-Look Laparoscopy

**DOI:** 10.1155/2016/3029264

**Published:** 2016-01-24

**Authors:** Matthias Korell, Nicole Ziegler, Rudy Leon De Wilde

**Affiliations:** ^1^Department of Obstetrics and Gynecology, Johanna-Etienne-Hospital, 41462 Neuss, Germany; ^2^Teaching Hospital of Heinrich Heine-University Duesseldorf, 40225 Düsseldorf, Germany; ^3^Clinic of Gynecology, Obstetrics and Gynecological Oncology, University Hospital for Gynecology, Pius Hospital Oldenburg, Medical Campus University of Oldenburg, Germany

## Abstract

*Purpose*. This study evaluates both scopes of 4DryField PH, certified for adhesion prevention and hemostasis, in patients undergoing surgery for various and severe gynecological disorders.* Methods*. This is a two-institutional study. Adhesion prevention efficacy was evaluated using video documentation of first-look laparoscopies (FLL) and second-look laparoscopies (SLL); other patient data were analyzed retrospectively. Twenty patients with various disorders were evaluated, 4 assigned to a uterus pathology, 10 to endometriosis, and 6 to an adhesion disease group. Nine patients received 4DryField primarily for hemostasis and 11 solely for adhesion prevention. Nineteen patients had SLL after 5 to 12 weeks and one after 13 months.* Results*. At FLL with 4DryField, immediate hemostasis could be achieved in diffuse bleeding. At SLL, effective adhesion prevention was observed in 18 of all 20 women, with only 2 revealing major adhesions. In particular, only 1 of the 6 women with adhesion disease as predominant disorder showed major adhesions at SLL.* Conclusions*. Modified polysaccharide 4DryField is not only effective in diffuse bleeding. In this cohort with extensive surgery for various gynecological pathologies, 4DryField showed effective adhesion prevention as confirmed at SLL, too. Its use as premixed gel is a convenient variant for treatment of large peritoneal wounds.

## 1. Introduction

The decision to use devices for adhesion prevention originates from the knowledge that postoperative adhesion formation occurs in up to >90% of the patients [1, 2]. In a considerable percentage, these adhesions cause a broad spectrum of complications, ranging from chronic pain to secondary female infertility to the severe complication of ileus with a significant mortality rate [3, 4]. This is leading to a high incidence of readmissions and repeated surgeries due to postoperative adhesions [5, 6]. Accordingly, there is a need for effective measures to reduce their high incidence [7].

It is determined within the first few days after surgery if the peritoneum heals with or without adhesions [8]. In contrast to, for example, skin lesions, the size of the peritoneal injury is secondary since even large defects regain a sufficient mesothelial coverage within this short period of time [9–11].

Many attempts have been undertaken to reduce the problem of postoperative adhesions. Only temporary barriers placed on such intra-abdominal wound surfaces and having a residence time corresponding with the short period of peritoneal regeneration are proven to be effective in reducing the still significantly impending complications of adhesions [12, 13].

Additionally, adhesions might be induced by residual intra-abdominal blood since their fibrin fibers can constitute bands connecting peritoneal surfaces and acting as a basis for adhesion strings [14, 15]. Thus, proper control of bleeding remains obligatory and sufficient hemostasis is considered as an important part of adhesion prevention.

While the hemostatic effect of a device can be assessed immediately on site, the adhesion prevention aspect can be valued only in the intermediate or later course. Additionally, the judgment is difficult since, although up to more than 90% of patients develop adhesions, not all of them experience complications [1, 2, 4]. Thus, the most compelling verification for efficiency of an adhesion prevention device might be demonstrated in patients who have an indication for a second-look laparoscopy (SLL).

Adhesions also play a central role in women with surgery for extended endometriosis since they are known to occur frequently and to be a cause for secondary female infertility [3, 6, 7, 16]. Thus, the proof that adhesions have not developed postoperatively is essential.

With 4DryField PH (PlantTec Medical GmbH, Bad Bevensen, Germany), a product certified for both adhesion prevention and hemostasis is available on the market. The efficacy of the product in the field of adhesion prevention has been shown in experimental studies [17, 18].

This clinical study was conducted to evaluate whether the experimental adhesion prevention results could be reproduced in surgery, particularly gynecological surgery. Since adhesion prevention basically can only be approved by second-look visual control, this study includes only patients in whom second-look laparoscopy was performed.

## 2. Patients and Methods

For this study, the data of 20 women (17–64 years, mean age 35.0 years), who had gynecological surgery in two separate institutions, were retrospectively evaluated. The initial intervention in context with this study was defined as first-look surgery. This also accounts for the 7 patients who had had previous surgeries.  Table 1 shows patients' demographics and baseline characteristics. It also can be derived from Table 1 that most of the women revealed several pathologies in need for treatment. Thus, to clarify, patients were assigned to groups according to their predominant disease: (I) uterus pathology group, (II) endometriosis group, and (III) adhesion disease group (Table 1).

All interventions, including the second-looks, were performed between September 2012 and April 2015. Patients consented to the application of the product, publication of their data and, in particular, intraoperative laparoscopic photographs not allowing to refer to patient's identity.

Table 2 summarizes pathologies and their extent of all 20 patients. The indications for first-look surgery in this study were abdominal or pelvic pain (*n* = 16), dysmenorrhea (*n* = 6), and the wish to conceive (*n* = 16).

Of the patients with primary uterus pathology, one had an endometrial carcinoma: she underwent total hysterectomy including bilateral salpingo-oophorectomy and pelvic lymphadenectomy. Two patients revealed multiple (up to 13) myomata, some with diameters of up to 12 cm. The remaining patient had an adenomyoma. Table 2 summarizes the pathologies and their extent.

All 10 patients assigned to endometriosis group revealed deep infiltrating disease. Resection resulted in large areas of peritoneal defects. Involvement of further structures outside the reproductive organs is indicated in Table 2. Five of 10 patients required excision of large bowel and/or rectal endometriosis. Three patients had involvement of ureter and two had involvement of the vagina. In 3 women additionally myomata had to be extirpated. Furthermore, all patients in this group showed various degrees of adhesion formation necessitating dissolution. All endometriosis patients showed an extensive degree of endometriosis disease often combined with other gynecological pathologies.

All 6 patients (Table 2) assigned to the adhesion disease group had a long history of severe symptoms ranging from abdominal-pelvic pain to intestinal obstruction. Four of the women had had multiple previous laparotomies and/or laparoscopies (1 of them even twice because of ileus). Only 1 woman had no previous surgery, and 1 patient had had a single previous surgery.

At first-look surgical exploration in the present study, all patients revealed extensive adhesion bands, interagglutination of intestinal loops, and/or bonding of intestinal loops to the abdominal wall. Additionally, all patients showed adhesion formation of the pelvic organs (including 16 women with wish to conceive). The extensive pathologies of the adhesion group are shown in Table 2.

All patients underwent only laparoscopic surgery and had 4DryField application at the end of surgery. The amount of 4DryField applied varied from 3 g to 15 g per patient.

In nine patients with an oozing wound ground, 4DryField was applied at first for hemostasis. The hemostatic efficiency of 4DryField powder was judged by subjective assessment of the surgeon. After complete hemostasis had been achieved, the remaining 4DryField powder was dripped with 0.9% saline solution. In doing so, a complete gel layer free of remnants of blood and protein was formed, providing the basis for adhesion prevention.

In the 11 further patients, 4DryField was applied solely for adhesion prevention. In these patients, the visceral side of areas depleted from peritoneum was covered with a layer of 4DryField. Subsequently, the powder was dripped with 0.9% saline solution until an area-wide gel layer had been formed. The parietal side was not covered with 4DryField, since after release of the pneumoperitoneum the gel layer of the viscera simultaneously acted as a sufficient barrier against the abdominal wall. In 2 instances, 4DryField was premixed in a kidney basin by the scrub nurse with saline solution (5 g 4DryField plus 40 mL of 0.9% saline solution). Subsequently, the gel was aspirated into a 100 mL syringe. 4DryField gel was applied directly to the viscera using a catheter.

At the end of each surgery, a drain was inserted in the pouch of Douglas. Patient records were evaluated concerning quantitative parameters of peripheral blood (hemoglobin, leucocytes, and C-reactive protein), temperature, stay of drains, and in-hospital stay of patients. Special attention was paid to any adverse events.

All second-look procedures were performed laparoscopically, in 19 patients 5 to 12 weeks after the first-look surgery and in 1 patient after 13 months. The latter patient had undergone surgery for endometrial carcinoma. Due to free fluid in the abdomen with positive cytology, she had second-look surgery for tumor staging.

Adhesions were assessed from both surgical reports and intraoperative video recordings and classified as 0 (no adhesions), I (minor filmy adhesions), and II (major adhesions), according to Corson et al. [19].

## 3. Results

### 3.1. First-Look Surgery

The pathologies of all patients at first-look constituting the basis for a comparison with second-look results are compiled in Table 2. In the 9 patients in whom 4DryField was administered primarily for diffuse oozing, the hemostatic efficiency of 4DryField was judged by subjective assessment of the surgeon. With application of the powder, immediate hemostasis could be observed. The use of further hemostatic adjuncts was not necessary; there was no conversion from laparoscopy to laparotomy. Postoperative transfusions were not necessary. Hemoglobin levels did not fall below 10 g/L. In two women, the C-reactive protein level exceeded 10 mg/dL (normal value <0.5 mg/dL), which was accompanied by mild leukocytosis in one of them (14.0/nL) but without elevated temperatures in both. Other incidences referring to adverse events due to the application of 4DryField were not found in any documentation.

Discharge from hospital was at day 4.5 ± 1.6 (2 to 7), comparable to other similar surgeries in Germany. Patients were free of pain without necessity of higher medication as usual for pain relief. There were no local infections: all wounds healed per primam.

### 3.2. Second-Look Surgery

At second-look laparoscopy, none of the four patients operated for uterus pathology had major adhesion formation. Only two patients revealed local adhesion formation at the posterior wall of the uterus or left ovary, which were classified as minor. The patient with endometrial carcinoma revealed neither adhesions in the area of hysterectomy or both iliac lymphadenectomies nor local recurrence of tumor in the pelvis; unfortunately, there were tumor metastases predominantly in the upper peritoneal cavity. Taking into account the extensive surgical measures at first-look intervention, overall adhesion formation was considered little during second-look surgery (Table 2).

**Figure 1:**
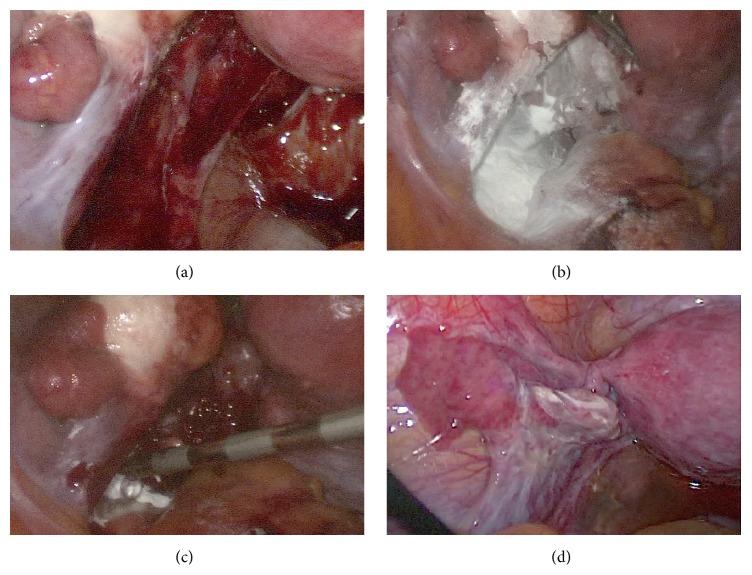
Pictures of surgery for endometriosis resection and site at second-look. (a) Area below left ovary after endometriosis resection from ovarian fossa to pouch of Douglas. (b) Wound surfaces of resection covered with 4DryField powder. (c) Transformation of 4DryField powder into a gel using 0.9% saline solution. (d) Site at second-look laparoscopy.

All 10 patients in the endometriosis group had deep infiltrating endometriosis. In 9 of them, 4DryField was used for hemostasis and adhesion prevention. Second-look laparoscopy revealed that in all but one patient areas treated with 4DryField showed no or minor adhesion formation. One patient had developed major adhesion bands in the resection area.  Figure 1 shows representative images of a surgery for endometriosis resection and successful prevention of adhesions.  Figure 1(a) shows the site after deeply infiltrating endometriosis below the left ovary extending from the ovarian fossa to the pouch of Douglas had been resected. In Figure 1(b), 4DryField powder is applied to the wound surfaces, and in Figure 1(c), the powder is transformed into a gel using 0.9% saline solution.  Figure 1(d) shows the site at second-look laparoscopy. The area of treatment (ovarian fossa and pouch of Douglas) is free of adhesions 7 weeks postoperatively. Adhesion formation results in all endometriosis patients can be found in Table 2. In summary, it can be quoted that the areas treated with 4DryField revealed no or minor adhesions in all but one patient.

In this study, the course of the 6 patients assigned to the adhesion group was of particular interest. In their medical history, 4 of these women had had multiple operations for recurrent adhesions, 1 of them even twice for ileus due to adhesions. As a first result, it could be deduced that this group consists of individuals who not only develop adhesions but also generate severe adhesion-related complications.

Remarkably, at second-look laparoscopy, only 1 of the 6 patients had recurrence of severe adhesions. Two patients had minor adhesions and three even had no adhesions. All 6 patients, including the one with recurrent adhesions, were free of pain.

**Figure 2:**
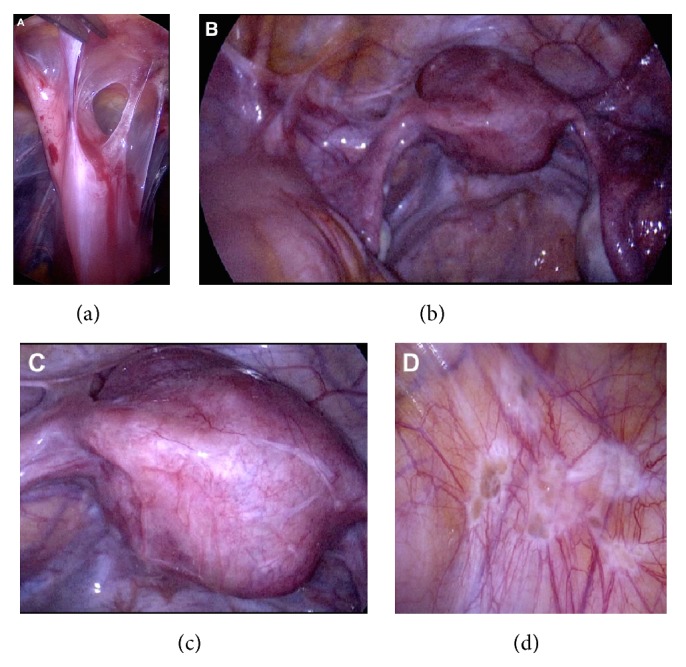
Various disorders in a patient of the adhesion disease group. (a) Adhesions between one of her laparotomy scars and small intestine. (b) Adhesions in the area of the pelvic organs. (c) Uterus with two myomata. (d) Endometriotic disease in the peritoneum of the abdominal wall.

**Figure 3:**
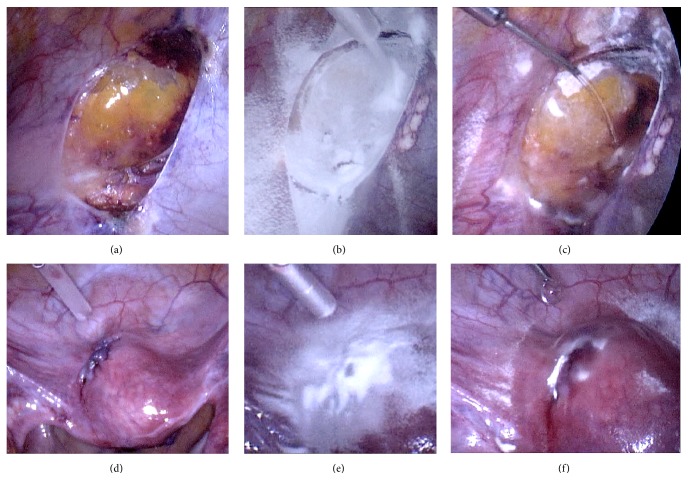
Treatment of the same patient as in Figure 2 with 4DryField. (a) Peritoneal defect after resection of endometriosis. (b) Application of 4DryField powder. (c) Transformation of the powder into a gel by dripping with 0.9% saline solution. (d) Sutures of myoma resection. (e) Treatment with 4DryField powder. (f) Dripping of powder with 0.9% saline solution to achieve gel formation.

**Figure 4:**
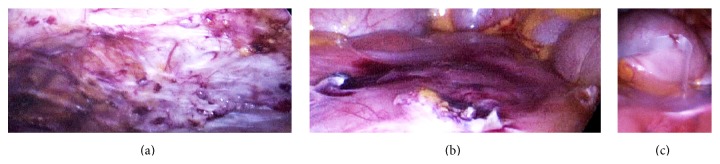
Treatment of the same patient as in Figures 2 and 3 with 4DryField. Extensive peritoneal defects after intestine adhesiolysis of the abdominal wall (a) and the intestine (b). (c) Application of premixed 4DryField gel on the vast wound areas of the intestine.

**Figure 5:**
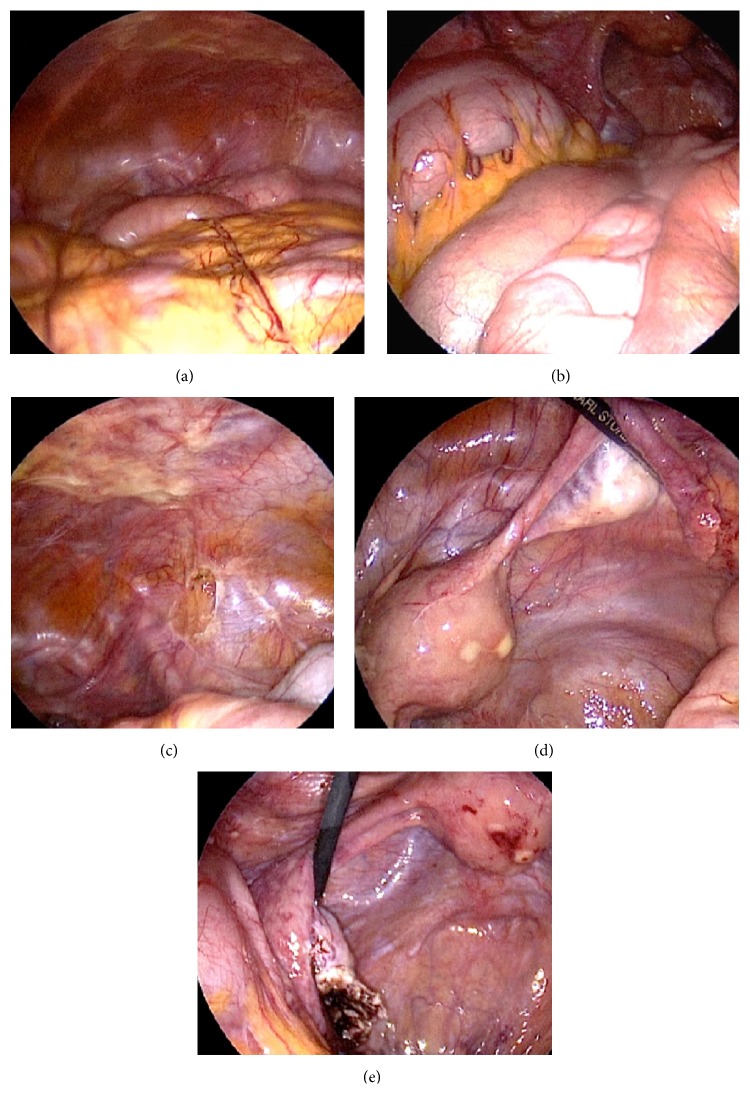
Operative site of the same patient as in Figures 2, 3, and 4 during second-look laparoscopy 5 weeks postoperatively. The intestine (a) and colon/sigma (b) are free of adhesions. (c) The wound in the abdominal wall left after endometriosis resection has healed. (d) and (e) The uterus and adnexa are free of adhesions and the wounds left after resection of uterine myomata have healed. (e) A large cyst removed from the left ovary during second-look.

Figures 2–5 show representative surgical images of the 43-year-old woman who in her case history had had multiple previous operations including two surgeries for ileus.

Figures 2, 3, and 4 are photographs taken during primary surgery, where the patient presented with extensive adhesion formation of small intestine, sigma/colon, and pelvic organs.  Figure 2(a) shows adhesions between one of her laparotomy scars and small intestine. Additionally, there were adhesions in the area of the pelvic organs (Figures 2(b) and 2(c)). Furthermore, there were 2 myomata (Figure 2(c)) as well as areas with active endometriotic disease (Figure 2(d)).

Figures 3 and 4 demonstrate the treatment with 4DryField.  Figure 3(a) shows the peritoneal defect after resection of endometriosis, Figure 3(b) the application of 4DryField as a powder, and Figure 3(c) the dripping on the powder with 0.9% saline solution to form a viscous gel to treat the peritoneal defect. The sutures of myoma resection (Figure 3(d)) were covered with 4DryField in the same fashion. Powder was applied on the suture line (Figure 3(e)) and then this was dripped with 0.9% saline solution to achieve gel formation (Figure 3(f)).

Treating the extensive peritoneal defect after intestinal adhesiolysis, 4DryField was applied as a premixed gel. In Figures 4(a) and 4(b), the extensive peritoneal defects of the abdominal wall (Figure 4(a)) and the intestine (Figure 4(b)) are visualized.  Figure 4(c) shows the application of premixed 4DryField gel on the wound areas of the intestine; the surface of the abdominal wall was not treated. The gel applied on the intestine formed a sufficient barrier between both visceral and parietal peritoneum upon release of pneumoperitoneum.

Figure 5 displays the operative site during second-look surgery 5 weeks postoperatively. The intestine (Figure 5(a)) and colon/sigma (Figure 5(b)) are completely free of adhesions. Additionally, the wound in the abdominal wall left after endometriosis resection has healed and shows the shiny surface of normal mesothelium (Figure 5(c)). The uterus and adnexa (Figures 5(d) and 5(e)) are free of adhesions. The wound surfaces left after resection of uterus myomata have healed without any adhesion formation (Figures 5(d) and 5(e)). During the second-look, a large cyst was removed from the left ovary (Figure 5(e)). In summary, for this patient with various pathologies and a long history of surgeries, the result is remarkable. Considering all patients of the adhesion disease group, in 5 of 6 women with 4DryField, effective results were found despite extensive primary surgical procedures (Table 2).

Summarizing the results of all 20 patients, the incidence of adhesion formation was remarkably low with 11 of 20 patients being completely free of recurring adhesions, 7 having developed minor, nonvascularized adhesions, and only 2 showing major adhesions. Thus, in these mostly multimorbid patients with various pathologies, after 4DryField treatment, promising results of adhesion prevention could be established in a high percentage (90%) of cases.

## 4. Discussion

The rate of adhesion formation after extensive surgery is reported to be up to >90% [1–3, 7]. There is broad consensus that adhesions form or do not form within the first few days postoperatively. Other than in skin, peritoneal healing is independent of the size of the defect. As soon as, postoperatively, a complete peritoneal coverage has been reconstituted, further adhesion formation is unlikely [8–11]. The introduction of laparoscopy is reported to reduce incidence and severity of adhesion formation as compared to laparotomy [20–22]. Despite that, neither complications nor costs have been reduced substantially by introducing laparoscopic surgery [23]. Since adhesions have a substantial clinical impact, much effort has been put into developing effective adhesion prevention devices in the last decades [24]. Although manifold devices for adhesion prevention are available, no single approach has been shown to be entirely successful [25–28]. In particular, since adhesions are the most frequent cause for secondary female infertility [16] and are a huge burden for patients with recurrent symptomatic adhesion formation [29], the search for effective devices for adhesion prevention remains essential. In the present study, this is reflected by the high proportions of women with wish to conceive (*n* = 16) and having severe symptomatic adhesions (*n* = 6).

Correspondingly, 3 of the 4 patients with primary uterus pathology wanted to become pregnant. In the dilemma that myoma surgery might induce the next and an even more severe cause for persisting secondary female infertility, the results with all 3 women being without major adhesions at second-look are promising.

The vicious circle that surgery-induced adhesions could cause infertility even more frequently than the primary disease itself [16] also accounts for all 10 patients assigned to the endometriosis group. In all instances, endometriosis had deeply infiltrated the peritoneum at various locations; thus, the risk for adhesion formation was particularly high in this cohort. Remarkably, 9 of 10 patients had no or only minor adhesions in the zones of 4DryField application; only one had major adhesions. Considering the severity of pathologies, this result can also be rated to be promising.

The 6 patients of the adhesion disease group were of particular interest since most of them had had a long history of previous interventions. At least the 4 patients with multiple previous surgeries can be characterized to be predestinated not only for recurrent adhesion formation but also for generating symptoms from their adhesions. Furthermore, the recurrence of adhesions after adhesiolysis is reported to be 55–100%, which also probably accounts for the laparoscopic approach [30–32]. In the underlying study, remarkably, 5 of the 6 patients of the adhesion disease group revealed no or minor, nonvascularized adhesions at second-look. Only 1 patient showed reformation of adhesions, albeit free of clinical symptoms. These results indicate that 4DryField is an efficient medical device for adhesion prevention, even in cases with recurrent adhesion disease.

Hemostats are not generally used in gynecological surgery but are only applied based on individual decision making by the surgeon. The subjective assessment of the efficiency was positive in all 9 patients in whom 4DryField was given for hemostasis. Besides reducing blood loss, sufficient hemostasis is one important condition in adhesion prevention. The latter is essential for long-term success.

Since adverse events did not occur, the modified polysaccharide can be assumed to be safe. The incidences with temporarily elevated C-reactive protein levels not accompanied by leukocytosis or fever can be interpreted to be due to the metabolization of 4DryField particles.

The combination of 4DryField powder serving as a hemostat and providing adhesion prevention when transformed into a gel is intriguing. The promising results of this cohort of patients comprising a variety of gynecological disorders correspond with the experimental results of Poehnert et al. [17, 18]. Despite extensive pathologies, only 2 of 20 patients developed major adhesions. In contrast, the other 18 patients showed no or few, nonvascularized adhesions at second-look laparoscopy. Further prospective and randomized studies in patients with high probability for adhesion formation are necessary to support these promising results.

## 5. Conclusion

With the use of the modified polysaccharide 4DryField, an immediate hemostatic effect could be observed in persisting oozing after resection of endometriosis, confirming its hemostatic capability.

In the cohort of women with various and severe gynecological disorders, 4DryField gel provides promising adhesion prevention; 18/20 women revealed no or minor adhesions as confirmed by second-look surgery performed in all cases. This also accounts for 5/6 women operated for severe symptomatic adhesion disease, that is, patients commonly known to suffer from their adhesions and with high probability of recurrence.

The use of 4DryField as a premixed gel has emerged as a convenient variant in the treatment of large areas of denudated peritoneum.

## Figures and Tables

**Table 1:** Demographics and baseline characteristics of all 20 patients.

	Mean age	Age range	Total	Myoma	Endometriosis	Adhesions
Uterus pathology group	41.5	29–64	4	4	1	4
Endometriosis group	30.7	23–39	10	3	10	10
Adhesion disease group	37.7	17–50	6	1	2	6

**Table 2:** Pathologies, involvement of other organs, and clinical outcome for all 20 patients. Adhesions at second-look were classified as 0 (no adhesions), I (minor filmy adhesions), and II (major adhesions), Corson et al. [19].

Uterus pathology group												

Mean age/ range	Total	Wish to conceive	Uterus pathology			Other pathologies		Clinical outcome		Adhesions at 2nd look		
			Multiple myoma	Adenomyoma	Endometrial carcinoma	Endometriosis resection	Adhesiolysis	Recurrent myoma	Peritoneal carcinosis	0	I	II

41.5 (29–64)	4	3	4	1	1	1	4	1	1	**2 (50%)**	**2 (50%)** ^1^	**0**

Endometriosis group												

Mean age/ range	Total	Wish to conceive	Organs involved			Other pathologies		Clinical outcome		Adhesions at 2nd look		
			Vagina	Rectum	Ureter	Myoma resection	Adhesiolysis	Free of pain	Remaining endometriosis	0	I	II

30.7 (23–39)	10	10	2	5	3	3	10	10	2	**6 (60%)**	**3 (30%)**	**1 (10%)**

Adhesion disease group												

Mean age/ range	Total	Wish to conceive	Organs involved			Other pathologies		Clinical outcome		Adhesions at 2nd look		
			Sigma/colon	Small intestine	Pelvic organs	Myoma resection	Endometriosis resection	Free of pain		0	I	II

37.7 (17–50)	6	3	6	6	6	1	2	6		**3 (50%)**	**2 (33%)**	**1 (17%)**

										Total		
										0	I	II

										**11 (55%)**	**7 (35%)**	**2 (10%)**

^1^One patient with one singular adhesion band, otherwise free of adhesions.
